# Allergies and Risk of Head and Neck Cancer: An Original Study plus Meta-Analysis

**DOI:** 10.1371/journal.pone.0055138

**Published:** 2013-02-01

**Authors:** Jenn-Ren Hsiao, Chun-Yen Ou, Hung-I Lo, Cheng-Chih Huang, Wei-Ting Lee, Jehn-Shyun Huang, Ken-Chung Chen, Tung-Yiu Wong, Sen-Tien Tsai, Chia-Jui Yen, Yuan-Hua Wu, Wei-Ting Hsueh, Ming-Wei Yang, Shang-Yin Wu, Jang-Yang Chang, Kwang-Yu Chang, Chen-Lin Lin, Fang-Ting Wang, Yi-Hui Wang, Ya-Ling Weng, Han-Chien Yang, Jeffrey S. Chang

**Affiliations:** 1 Department of Otolaryngology, National Cheng Kung University Hospital, College of Medicine, National Cheng Kung University, Tainan, Taiwan; 2 Department of Stomatology, National Cheng Kung University Hospital, College of Medicine, National Cheng Kung University, Tainan, Taiwan; 3 Division of Hematology/Oncology, Department of Internal Medicine, National Cheng Kung University Hospital, College of Medicine, National Cheng Kung University, Tainan, Taiwan; 4 Department of Radiation Oncology, National Cheng Kung University Hospital, College of Medicine, National Cheng Kung University, Tainan, Taiwan; 5 National Institute of Cancer Research, National Health Research Institutes, Tainan, Taiwan; 6 Department of Nursing, National Cheng Kung University Hospital, College of Medicine, National Cheng Kung University, Tainan, Taiwan; Baylor College of Medicine, United States of America

## Abstract

**Background:**

Although the relationship between allergy and cancer has been investigated extensively, the role of allergy in head and neck cancer (HNC) appears less consistent. It is not clear whether allergies can independently influence the risk of HNC in the presence of known strong environmental risk factors, including consumption of alcohol, betel quid, and cigarette.

**Methods:**

The current paper reports results from: 1) an original hospital-based case-control study, which included 252 incident cases of HNC and 236 controls frequency-matched to cases on sex and age; and 2) a meta-analysis combining the results of the current case-control study and 13 previously published studies (9 cohort studies with 727,569 subjects and 550 HNC outcomes and 5 case-control studies with 4,017 HNC cases and 10,928 controls).

**Results:**

In the original case-control study, we observed a strong inverse association between allergies and HNC [odds ratio = 0.41, 95% confidence interval (CI): 0.27–0.62]. The meta-analysis also indicated a statistically significant inverse association between HNC and allergies [meta-relative risk (RR) = 0.76, 95% CI: 0.63–0.91], particularly strong for allergic rhinitis (meta-RR = 0.55, 95% CI: 0.40–0.76). In addition, the inverse association between allergies and HNC was observed only among men (meta-RR = 0.67, 95% CI: 0.54–0.84) but not among women (meta-RR = 0.98, 95% CI: 0.81–1.18).

**Conclusions:**

These findings suggest that immunity plays an influential role in the risk of HNC. Future studies investigating immune biomarkers, including cytokine profiles and genetic polymorphisms, are warranted to further delineate the relationship between allergies and HNC. Understanding the relationship between allergies and HNC may help devise effective strategies to reduce and treat HNC.

## Introduction

Head and neck cancer (HNC), including cancers of the oral cavity, oropharynx and larynx, is one of the leading cancers worldwide. Each year, approximately 400,000 cases of oral and oropharyngeal cancer and 160,000 cases of laryngeal cancer are diagnosed worldwide [1]. HNC is also a leading cancer among Taiwanese men, who have the second highest incidence of HNC in the world [2]. According to the Department of Health of Taiwan, HNC was the fourth most common cancer among Taiwanese men in 2009, with an annual incidence of 41 per 100,000 persons [3].

The occurrence of most HNC can be attributed to the consumption of alcohol, cigarette, and betel quid [4], [5], although human papillomavirus infection also plays a role, particularly for oropharyngeal cancer [6]. Although alcohol, cigarette, and betel quid are the causes for the majority of the HNC cancer cases, most people who consume these three agents will not develop HNC in their lifetimes, which suggests the roles of other environmental and genetic factors [7], [8]. Other potential risk factors for HNC include occupational exposures, poor oral hygiene, mouthwashes containing alcohol, and low fruit and vegetable intake [1], [9]; however, there is insufficient knowledge regarding the pathogenesis of HNC.

Immune reactions manifested in the form of allergies have been extensively studied for their relationship with cancer risk [10]–[12]. Allergies usually result from an overactive immune response to substances in the environment among people with atopic tendency, who are genetically predisposed to produce immunoglobulin E (IgE) against common allergens [13]–[15]. The cross-linking of IgE on the surface of mast cells and basophils leads to a series of events resulting in allergic diseases, including allergic rhinitis, asthma, eczema, and food allergy [14].

Two hypotheses have been proposed to explain the inverse association between allergies and cancer, the “immunosurveillance hypothesis” and the “prophylaxis hypothesis” [11]. In the “immunosurveillance hypothesis”, allergy does not play a direct role to reduce the risk of cancer but is merely a side effect of a more active immune function that effectively detects and eradicates malignant cells [11]. In contrast, allergy plays a direct role to reduce the risk of cancer in the “prophylaxis hypothesis” by expelling toxins or pathogens from the body [11]. A reduced risk associated with allergies has been consistently observed for glioma and pancreatic cancer [16], [17], two cancers with largely unknown environmental risk factors. Compared to glioma and pancreatic cancer, the role of allergy in HNC appears less consistent. It is not clear whether allergies can independently influence the risk of HNC in the presence of known strong environmental risk factors, including alcohol, betel quid, and cigarette. In addition, only one previous study has examined the interaction between lifestyle factors (cigarette smoking and alcohol drinking) and allergies on the risk of HNC [18]. To this end, we conducted a hospital-based study to examine the association between allergies and HNC, including the interaction between allergies and alcohol, betel quid, or cigarette on the development of HNC. In addition, a comprehensive literature search was performed to conduct a meta-analysis, combining results from the published literature with the current study in order to explore the different aspects of the relationship between allergies and HNC and to generate new hypotheses for further investigations.

## Materials and Methods

### Original Study

This hospital-based case-control study was approved by the institutional review boards of the National Cheng Kung University Hospital and the National Health Research Institutes. All participants of the study signed a written informed consent.

#### Study subjects

All of the study subjects were recruited from the Department of Otolaryngology and the Department of Stomatology at the National Cheng Kung University Hospital from September 1, 2010 to June 30, 2012. The case subjects were patients newly diagnosed with HNC, including cancers of the oral cavity, oropharynx, and larynx, had no cancer history, aged 20 to 80 years, and had the ability to give informed consent. All HNC cases were pathologically confirmed squamous cell carcinoma. The controls subjects were patients who required surgery for non-cancerous diseases unrelated to cigarette smoking, alcohol drinking, or area nut/betel quid chewing, had no cancer history, had the ability to give informed consent, and frequency-matched to the cases on age (±5 years) and sex.

#### Data collection

In-person interview was conducted with each study subject by a trained interviewer using a standardized questionnaire. Demographic information, including sex, age, and educational level were collected. Detailed information (dose and duration) of the three major risk factors of HNC, including alcohol, betel quid, and cigarette, was ascertained. Study subjects were asked whether they had any of the five types of allergies, including allergic rhinitis, skin allergy, food allergy, drug allergy, and asthma. Those that responded positive were further asked about the age of onset for these allergies.

#### Statistical analysis

To assess for the differences in demographic variables and lifestyle habits (alcohol drinking, betel quid chewing, and cigarette smoking) between cases and controls, t-tests and chi-squared tests were performed for continuous variables and categorical variables, respectively. Multivariable unconditional logistic regression was performed to estimate the odds ratio (OR) and 95% confidence interval (CI) of HNC associated with allergies adjusted for age, sex, education, alcohol drinking (frequency), betel quid use (pack-years), and cigarette smoking (pack-years). Although, no pack-year definition has been established for betel-quid use, in order to adjust for the amount and the duration of betel quid use, a pack-year definition similar to the pack-year of cigarette of smoking was used, with 1 pack-year of betel quid use = 1 pack of betel quid (20 betel quids) use per day ×1 year. The analysis was first performed with all allergies combined (yes/no to any of the five allergies, number of allergies, duration of allergies, and age of allergic symptom onset). Subsequent analyses were performed to assess the association between HNC risk and individual types of allergies. To evaluate for the influence of allergy on the association between alcohol, betel quid, or cigarette and HNC, unconditional logistic regression was performed stratified by allergy status (yes/no). The heterogeneity of the association between alcohol, betel quid or cigarette and HNC risk by allergy status was assessed by the log-likelihood ratio test comparing the unconditional logistic regression model with the interaction term (allergy × alcohol, allergy × betel quid, or allergy × cigarette) to the model without the interaction term.

### Meta-analysis

#### Literature search

**Figure 1:**
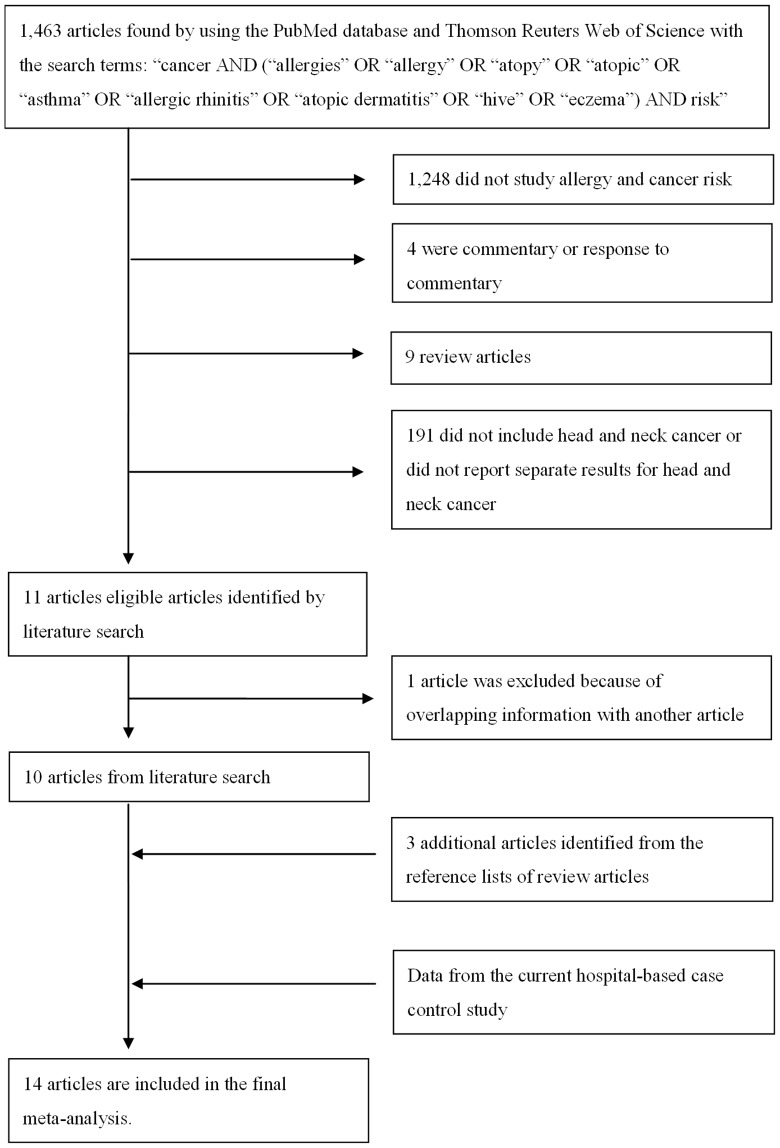
Process of article exclusion.

A literature search was conducted until July 18, 2012 using the PubMed database and Thomson Reuters Web of Science with the search terms: “cancer AND (“allergies” OR “allergy” OR “atopy” OR “atopic” OR “asthma” OR “allergic rhinitis” OR “atopic dermatitis” OR “hive” OR “eczema”) AND risk”. A total of 1,463 articles were identified using the search terms (Figure 1). Articles were excluded if they had the following characteristics: 1) did not study allergy and cancer risk (n = 1,248); 2) commentary or response to commentary (n = 4); 3) review articles (n = 9); or 4) did not include HNC or did not report separate results for HNC (n = 191). Eleven articles were identified from this initial literature search.[18]–[28] No additional articles were identified by reviewing the reference lists of the 11 articles identified from the literature search. Two articles by Ericsson had overlapping information and only the most recent article was included in the current meta-analysis [21], [22]. Three more articles [29]–[31] were identified by searching through the reference lists of four review articles [10]–[12], [32]. Along with our original case-control study, a total of 14 studies (9 cohort studies with 727,569 subjects and 550 HNC outcomes and 5 case-control studies with 4,017 HNC cases and 10,928 controls) (Table S1) was included in the meta-analysis.

#### Data abstraction and calculation of summary statistics

The data extracted from each article included authors, year of publication, types of study (cohort or case-control studies), number of study subjects, variables used for matching and for statistical adjustments, and relative risk (RR, which includes incidence ratio or standardize mortality ratio for cohort studies and odds ratio for case-control studies) with corresponding 95% CIs. For studies that presented only frequency data, RRs and 95% CIs were calculated using the exact method. If a study only reported RRs separately by gender, specific types of allergy or HNC sites, a meta-analysis with a fixed effects model was performed to combine stratum-specific RRs into an overall RR of HNC.

#### Statistical analysis

STATA software (version 11.0; StataCorp, College Station, TX, USA) was used to perform analyses of the current meta-analysis. Summary RRs (meta-RRs) was generated by the “metan” command [33]. The RRs of individual studies are displayed in a forest plot. I^2^ statistic was used to evaluate inconsistencies between studies [34]. I^2^ = 0–24.9%, 25–49.9%, and 50% or more represent low, moderate and high heterogeneity between studies, respectively. The summary RR was generated by a random effects model using the DerSimonian and Laird method if the heterogeneity was moderate to high (I^2^≥25%) [35] and by a fixed effects model if the heterogeneity was low (I^2^<25%). The analysis was first performed to generate an overall meta-RR measuring the association between all allergies combined and HNC risk. Additional analyses were performed by study design (cohort vs. case-control), by sex, by HNC sites, and by specific types of allergies (allergic rhinitis, skin allergy, or asthma). Sensitivity analysis was performed using the “metainf” command by excluding one study at a time to examine whether any individual study had a dominant effect on the summary RR. Publication bias was examined by viewing the funnel plot and by Egger’s regression asymmetry test [36], [37].

## Results

### Original Study

From September 1, 2010 to June 30, 2012, 252 HNC cases (166 oral cancers, 61 oropharyngeal cancers, and 25 laryngeal cancers) and 236 controls were successfully recruited with a participation of 74.3% and 86.5% for the cases and controls, respectively. The HNC cases were slightly older than controls (55.3 years vs. 53.5 years, *P* = 0.05) (Table 1). HNC cases and controls were similar in the distribution of sex (Table 1). Compared to controls, cases had lower educational levels (37% with ≧ high school education among cases vs. 65% with ≧ high school education among controls, *P*<0.0001) (Table 1). Cases had higher consumptions of alcohol, betel quid, and cigarette compared to controls in frequency, duration, and amount (Table 1).

**Table 1:** Demographic and lifestyle characteristics of the head and neck cancer patients and control subjects.

Characteristics	Cases N = 252 n (%)	Controls N = 236 n (%)	*P*
**Age (years)**			
Mean (SE)	55.3 (0.7)	53.5 (0.7)	0.05
**Sex**			
Male	238 (94.4)	220 (93.2)	0.57
Female	14 (5.6)	16 (6.8)	
**Education**			
≦ Elementary school	81 (32.1)	40 (16.9)	<0.0001
Junior high	77 (30.6)	42 (17.8)	
High school/Technical school	74 (29.4)	96 (40.7)	
Some college or more	20 (7.9)	58 (24.6)	
**Alcohol**			
Never+occasional	69 (27.4)	100 (42.4)	0.002
Former regular	49 (19.4)	33 (14.0)	
Current regular	134 (53.2)	103 (43.6)	
Never	62 (24.9)	85 (36.2)	<0.0001
1 drink per month	14 (5.6)	29 (12.3)	
1–2 drinks per week	10 (4.1)	14 (6.0)	
3–5 drinks per week	21 (8.4)	31 (13.2)	
Daily drinkers	142 (57.0)	76 (32.3)	
**Betel quid**			
Never	90 (35.7)	181 (76.7)	<0.0001
Former	103 (40.9)	36 (15.2)	
Current	59 (23.4)	19 (8.1)	
Pack-years (SE)	27.3 (2.9)	7.1 (1.3)	<0.0001
**Cigarette**			
Never	35 (13.9)	66 (28.0)	0.0001
Former	56 (22.2)	58 (24.6)	
Current	161 (63.9)	112 (47.4)	
Pack-years (SE)	35.1 (1.8)	23.5 (1.6)	<0.0001

Abbreviation: N, number; SE, standard error.

Having any allergy was associated with a reduced risk of HNC (OR = 0.41, 95% CI: 0.27–0.62) (Table 2). This reduced risk was observed across all three HNC sites (oral cancer: OR = 0.36, 95% CI: 0.22–0.57; oropharyngeal cancer: OR = 0.49, 95% CI: 0.25–0.96; laryngeal cancer: OR = 0.48, 95% CI: 0.19–1.18). An inverse association between allergies and HNC was observed among both genders and was particularly strong for men (OR = 0.42, 95% CI: 0.27–0.64) but not statistically significant for women (OR = 0.67, 95 CI: 0.08–5.46), although the result for women was based on small numbers (14 cases and 16 controls). No clear trend in HNC risk was observed with the number of allergies or the duration of allergies (Table 2). In addition, the association between HNC risk and allergies did not differ by the age of allergy onset (<21 years vs. 21 years or older) (Table 2).

**Table 2:** The association between allergies and head and neck cancer.

Allergy	Head and neck cancer N = 252 n (%)	Control N = 236 n (%)	OR (95% CI)*	*P*
No	164 (65.1)	95 (40.3)	Referent	
Yes	88 (34.9)	135 (57.2)	0.41 (0.27–0.62)	<0.0001
Unknown	0 (1.2)	6 (2.5)	–	–
**Number of allergies**				
0	164 (65.1)	95 (40.2)	Referent	
1	54 (21.4)	80 (33.9)	0.39 (0.24–0.64)	0.0002
2	23 (9.1)	32 (13.6)	0.54 (0.28–1.02)	0.06
3 or more	10 (4.0)	17 (7.2)	0.40 (0.16–1.03)	0.06
unknown	1 (0.4)	12 (5.1)	–	–
**Allergy duration**				
No allergy	164 (65.1)	95 (40.3)	Referent	
<10 years	18 (7.1)	25 (10.6)	0.48 (0.23–0.99)	0.05
10–20 years	10 (4.0)	23 (9.7)	0.32 (0.14–0.75)	0.008
>20 years	48 (19.0)	67 (28.4)	0.45 (0.27–0.74)	0.002
Unknown	12 (4.8)	26 (11.0)	–	–
**Age of allergy onset**				
No allergy	164 (65.1)	95 (40.3)	Referent	
<21 years old	27 (10.7)	52 (22.0)	0.38 (0.21–0.68)	0.001
21 years or older	53 (21.0)	75 (31.8)	0.42 (0.26–0.69)	0.0005
Unknown	8 (3.2)	14 (5.9)	–	–

* OR was adjusted for age, sex, education, alcohol drinking (frequency), betel quid chewing (pack-years), and cigarette smoking (pack-years).

Abbreviations: CI, confidence interval; N, number; OR, odds ratio.

The most common type of allergy reported by the study subjects was allergic rhinitis (20% of cases and 41% of controls), which was associated with a reduced risk of HNC (OR = 0.32, 95 CI: 0.20–0.52) (Table 3). A consistent reduction in the risk of HNC was seen with the other four types of allergies, including skin allergy, food allergy, drug allergy, and asthma.

**Table 3:** The association between head and neck cancer and specific types of allergies.

Allergies	Cases N = 252	Controls N = 236	OR (95% CI)*	*P*
**Nasal allergy**				
No allergy	164 (65.1)	95 (40.2)	Referent	
Yes	51 (20.2)	97 (41.1)	0.32 (0.20–0.52)	<0.0001
Other allergies	36 (14.3)	36 (15.3)	0.67 (0.37–1.20)	0.18
Unknown	1 (0.4)	8 (3.4)	–	–
**Skin allergy**				
No allergy	164 (65.1)	95 (40.2)	Referent	
Yes	39 (15.5)	40 (17.0)	0.67 (0.38–1.17)	0.16
Other allergies	49 (19.4)	92 (39.0)	0.32 (0.20–0.52)	<0.0001
Unknown	0 (0.0)	9 (3.8)	–	–
**Food allergy**				
No allergy	164 (65.1)	95 (40.2)	Referent	
Yes	20 (7.9)	23 (9.8)	0.63 (0.30–1.31)	0.21
Other allergies	68 (27.0)	112 (47.5)	0.38 (0.24–0.58)	<0.0001
Unknown	0 (0.0)	6 (2.5)	–	–
**Drug Allergy**				
No allergy	164 (65.1)	95 (40.2)	Referent	
Yes	16 (6.3)	29 (12.3)	0.44 (0.21–0.92)	0.03
Other allergies	72 (28.6)	105 (44.5)	0.41 (0.27–0.64)	<0.0001
Unknown	0 (0.0)	7 (3.0)	–	–
**Asthma**				
No allergy	164 (65.1)	95 (40.2)	Referent	
Yes	7 (2.8)	17 (7.2)	0.26 (0.09–0.73)	0.01
Other allergies	81 (32.1)	118 (50.0)	0.44 (0.29–0.67)	0.0001
Unknown	0 (0.0)	6 (2.6)	–	–

* OR was adjusted for age, sex, education, alcohol drinking (frequency), betel quid chewing (pack-years), and cigarette smoking (pack-years).

Abbreviations: CI, confidence interval; N, number; OR, odds ratio.

The increased risk of HNC associated with alcohol drinking, betel quid chewing or cigarette smoking appeared stronger among those without history of allergies, although the tests for heterogeneity were not statistically significant (Table 4).

**Table 4:** The association between lifestyle factors and head and neck cancer by allergy status.

Characteristics	Allergy = Yes OR (95% CI)*	Allergy = No OR (95% CI)*	Interaction *P*
**Alcohol**			
Never+non-daily drinkers	Referent	Referent	
Ever daily drinker	1.47 (0.76–2.85)	2.65 (1.37–5.13)	0.17
Never+non-daily drinkers	Referent	Referent	
Former daily drinker	1.18 (0.40–3.51)	2.23 (0.62–8.10)	
Current daily drinkers	1.56 (0.77–3.18)	2.73 (1.37–5.45)	0.39
**Betel quid**			
Never	Referent	Referent	
Ever	4.25 (2.14–8.42)	5.62 (2.84–11.12)	0.49
Never	Referent	Referent	
Former	3.82 (1.82–7.99)	6.31 (2.88–13.82)	0.49
Current	5.48 (2.08–14.45)	4.57 (1.80–11.59)	
Pack-years			
Never	Referent	Referent	
<20 pack-years	3.29 (1.44–7.49)	3.97 (1.66–9.50)	
≧20 pack-years	4.76 (2.04–11.09)	6.51 (2.84–14.95)	0.80
**Cigarette**			
Never	Referent	Referent	
Ever	0.98 (0.41–2.38)	2.63 (1.17–5.93)	0.09
Never	Referent	Referent	
Former	0.87 (0.31–2.41)	2.60 (1.05–6.42)	0.28
Current	1.05 (0.42–2.62)	2.66 (1.11–6.39)	
Pack-years			
Never	Referent	Referent	
<20 pack-years	0.62 (0.21–1.77)	3.17 (1.15–8.73)	0.11
≧20 pack-years	1.28 (0.51–3.22)	(1.04–5.62)	

* OR was adjusted for age, sex, education, alcohol drinking (frequency), betel quid chewing (pack-years), and cigarette smoking (pack-years).

Abbreviations: CI, confidence interval; OR, odds ratio.

### Meta-analysis (Table 5)

**Figure 2:**
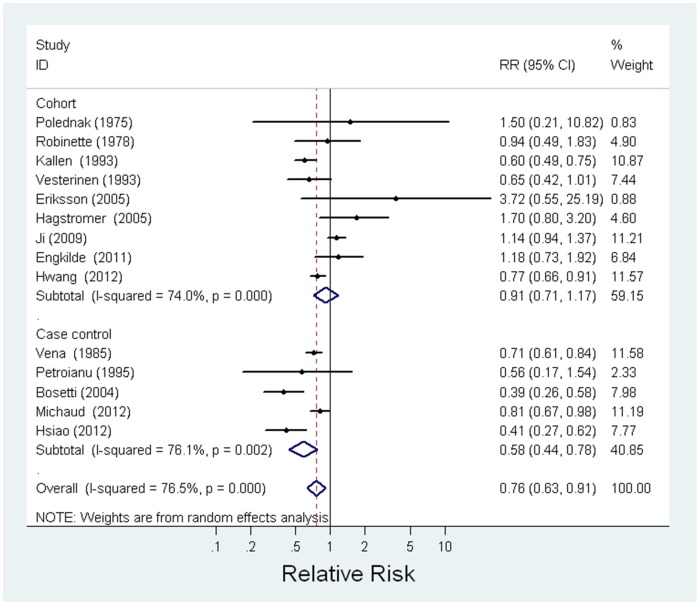
Meta-analysis assessing the association between allergies and head and neck cancer Risk.

**Figure 3:**
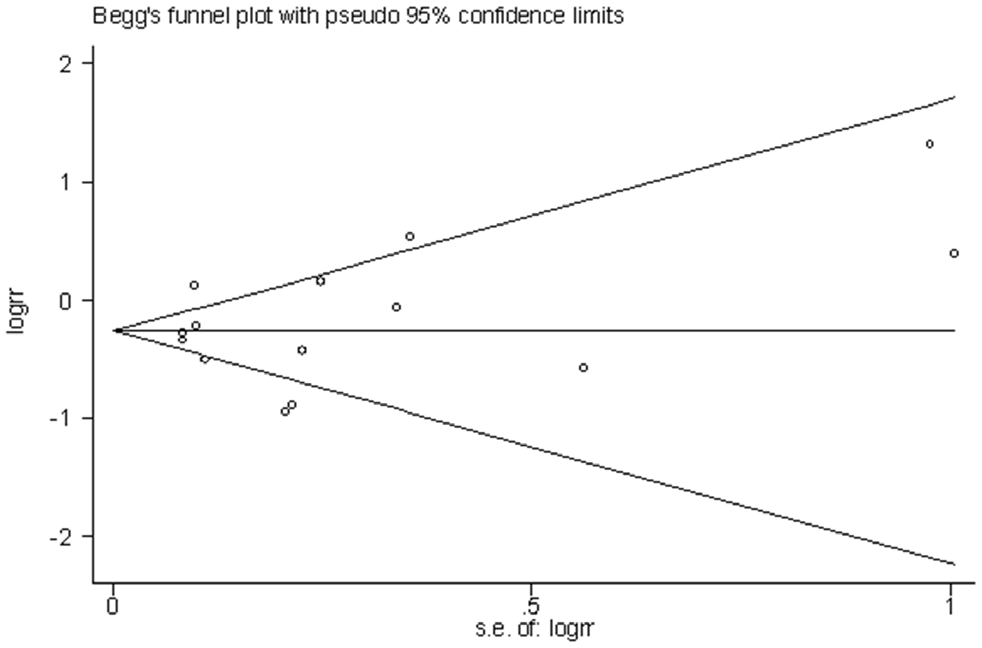
Funnel plot of the 14 studies assessing the association between allergies and head and neck cancer risk.

Combining the data from 14 studies (9 cohort and 5 case-control studies), the meta-analysis showed a reduced risk of HNC associated with having allergies (meta-RR = 0.76, 95% CI: 0.63–0.91, I^2^ = 76.5%) (Figure 2). Sensitivity analysis by dropping one study at a time did not indicate a dominant influence of any one individual study on the summary RR. The Egger’s regression asymmetry test indicated no evidence of publication bias (p = 0.07). Funnel plot (Figure 3) showed that there may be minor evidence of publication bias with a slight over-representation of smaller studies (n = 2) [22], [31] with a RR value greater than the meta-RR. However, the meta-analysis excluding those two smaller studies generated a result (meta-RR = 0.74, 95% CI: 0.62–0.90, I^2^ = 79.0%) that was nearly identical to the result that combined all 14 studies, which indicated that the possibility of publication bias was minimal.

The combined analysis of 9 cohort studies with 727,569 subjects with allergies and 550 HNC outcomes showed a 9% reduction in HNC risk associated with allergies, although not statistically significant (meta-RR = 0.91, 95% CI: 0.71–1.17, I^2^ = 74.0%). The combined analysis of 5 case-control studies with 4,017 HNC cases and 10,928 controls showed a statistically significant inverse association between allergies and HNC (meta-RR = 0.58, 95% CI: 0.44–0.78, I^2^ = 76.1%).

When stratified by sex, the inverse association between allergies and HNC was only observed among men (meta-RR = 0.67, 95% CI: 0.54–0.84, I^2^ = 68.2%) but not among women (meta-RR = 0.98, 95% CI: 0.81–1.18, I^2^ = 20.2%). In the analysis with specific types of allergies, the strongest reduced risk of HNC was observed for allergic rhinitis (meta-RR = 0.55, 95% CI: 0.40–0.76, I^2^ = 46.1%) and a weaker reduction in HNC risk was observed for skin allergy (meta-RR = 0.84, 95% CI: 0.62–1.14, I^2^ = 43.0%) and asthma (meta-RR = 0.86, 95% CI: 0.71–1.05, I^2^ = 63.1%). A reduced risk associated with allergies was observed across all three sites of HNC (meta-RR for oral cancer = 0.78, 95% CI: 0.61–1.00, I^2^ = 72.2%; meta-RR for oropharyngeal cancer = 0.69, 95% CI: 0.58–0.82, I^2^ = 33.6%; meta-RR for laryngeal cancer = 0.66, 95% CI: 0.47–0.92, I^2^ = 53.0%) (Table 5); however, similar to the results of all sites combined, the inverse association between allergies and subsites of HNC occurred predominantly among men.

**Table 5:** Meta-analysis on the association between allergy and head and neck cancer.

	Both genders combined			Male			Female		
	n	Meta-RR (95% CI)*	I^2^	n	Meta-RR (95% CI)*	I^2^	n	Meta-RR (95% CI)*	I^2^
**Head and neck cancer**									
All allergy combined	14	0.76 (0.63–0.91)	76.5	9	0.67 (0.54–0.84)	68.2	7	0.98 (0.81–1.18)	20.2
*Study designs*									
Cohort	9	0.91 (0.71–1.17)	74.0	5	0.80 (0.60–1.07)	34.7	3	0.89 (0.48–1.65)	47.2
Case–control	5	0.58 (0.44–0.78)	76.1	4	0.56 (0.39–0.81)	83.4	4	0.98 (0.79–1.21)	19.6
Allergic rhinitis	3	0.55 (0.40–0.76)	46.1	3	0.53 (0.42–0.66)	20.0	3	1.25 (0.77–2.04)	0.0
Skin allergy	5	0.84 (0.62–1.14)	43.0	4	0.82 (0.62–1.08)	0.0	3	0.78 (0.50–1.21)	0.0
Asthma	9	0.86 (0.71–1.05)	63.1	6	0.79 (0.61–1.02)	35.8	3	1.55 (1.02–2.38)	0.0
**Oral cancer**									
All allergy combined	9	0.78 (0.61–1.00)	72.2	7	0.64 (0.45–0.91)	69.1	4	1.06 (0.76–1.50)	33.7
Allergic rhinitis	3	0.56 (0.36–0.88)	66.6	3	0.48 (0.31–0.75)	48.8	2	1.31 (0.79–2.16)	0.0
Skin allergy	5	0.84 (0.52–1.34)	64.8	4	0.77 (0.48–1.25)	42.7	2	0.68 (0.42–1.12)	0.0
Asthma	6	0.88 (0.74–1.04)	0.0	5	0.76 (0.57–1.02)	0.0	2	1.59 (0.97–2.61)	0.0
**Pharynx**									
All allergy combined	10	0.69 (0.58–0.82)	33.6	7	0.67 (0.45–1.00)	61.4	4	1.12 (0.60–2.12)	49.5
Allergic rhinitis	3	0.43 (0.23–0.80)	37.1	3	0.49 (0.22–0.83)	41.7	0	–	–
Skin allergy	4	0.92 (0.65–1.30)	0.0	4	0.97 (0.62–1.50)	17.9	2	0.75 (0.33–1.74)	42.4
Asthma	7	0.67 (0.56–0.79)	0.0	5	0.79 (0.54–1.15)	0.0	2	2.06 (0.70–6.10)	0.0
**Larynx**									
All allergy combined	7	0.66 (0.47–0.92)	53.0	5	0.64 (0.42–0.96)	56.7	2	0.67 (0.22–2.04)	0.0
Allergic rhinitis	2	0.39 (0.13–1.13)	42.2	2	0.40 (0.15–1.08)	33.1	0	–	–
Skin allergy	2	1.07 (0.61–1.85)	0.0	2	1.10 (0.63–1.92)	0.0	0	–	–
Asthma	4	0.86 (0.58–1.28)	38.8	3	0.92 (0.48–1.74)	67.3	1	–	–

* The Meta-RR was generated by a random effects model using the DersSimonian and Laird method if the heterogeneity was high to moderate (I^2^≥25%) and by a fixed effects model if the heterogeneity was low (I^2^<25%).

Abbreviations: CI, confidence interval; meta-RR, meta-relative risk; n, number of studies.

## Discussion

A statistically significant inverse association between allergies and HNC was observed in our hospital-based case-control study. Combining the results of our study and those from the 13 published studies, the meta-analysis also indicated a statistically significant inverse association between HNC and allergies, particularly for allergic rhinitis and predominantly among men.

The inverse association between allergies and cancer may be explained by two hypotheses, the “immunosurveillance hypothesis” and the “prophylaxis hypothesis” [11]. In the “immunosurveillance hypothesis”, allergy is a side effect of an overactive immune function that effectively detects and eradicates malignant cells and does not play a direct role to reduce the risk of cancer [11]. In contrast, in the “prophylaxis hypothesis”, allergy is directly involved in cancer prevention by expelling toxins or pathogens from the body [11]. A review by Sherman *et al.* observed that the inverse association between allergies and cancer was more commonly found among tissues or organ systems that interface with the external environment (*i.e.* glia, mouth and throat, colon and rectum, pancreas, skin, uterus and cervix, lung, bladder, and gastrointestinal tract) than among those that do not (*i.e.* ovaries, meninges, prostate, breast, and blood cells); this led them to conclude that the “prophylaxis hypothesis” may be the more appropriate explanation for the site-specific inverse association between allergies and certain cancers [11]. Although the results from our case-control study and meta-analysis appear to be consistent with the “prophylaxis hypothesis” because the three sites of HNC, oral cavity, oropharynx, and larynx, directly interface with the external environment, the role of “immunosurveillance hypothesis” cannot be ruled out by the evidence from this analysis. Further investigation is needed to delineate the underlying biological mechanism for the inverse association between allergies and HNC risk.

IgE plays a key role in most allergic reactions and evidence shows that IgE itself may possess anti-tumor activities [38]. An immunohistochemical study by Neuchrist *et al.* examined the distribution of immunoglobulins in squamous cell carcinoma of head and neck by comparing tumor and normal tissues [39]. They found that the distribution of IgE showed the largest difference, with significantly higher number of IgE-positive cells found in the tumor tissue compared to the normal tissue [39], which suggested that IgE may participate in tumor surveillance. In addition, IgE may also bind to cell surface IgE receptors, FcεRI and CD23, and engage several types of effector cells in antibody-dependent cellular cytotoxicity and antibody-dependent cellular phagocytosis against cancer cells [38]. Overall, the possible anti-tumor activities of IgE may partially explain the inverse association between allergies and cancer, including HNC.

In our original case-control study, there was a suggestion that allergies may attenuate the carcinogenic effect of alcohol, betel quid, or cigarette on the development of HNC risk, although the results were not statistically significant likely due to a sample size without sufficient statistical power. A possible explanation for the attenuating effect of allergies on HNC risk due to alcohol, betel quid, or cigarette may be a hyperactive immune function that can detect and destroy malignant cells before they have a chance to grow and expand. Alternatively, allergy-associated immune activities may rid body of the carcinogens before they have a chance to induce the oncogenic transformation of normal cells. However, in a previous study by Michaud *et al.* the association between allergies and HNC risk did not differ by smoking status (never smoker: OR = 0.75, 95% CI:0.54–1.04; former smoker: OR = 0.80, 95% CI: 0.62–1.02) or drinking status (less than 7 drinks per week: OR = 0.78, 95% CI: 0.60–1.01; greater than 25 drinks per week: OR = 0.59, 95% CI: 0.37–0.95) [18]. Unfortunately, among the studies to date, only Michaud *et al*. [18] and our study had examined the interaction between allergies and lifestyle factors (cigarette smoking, alcohol drinking, or betel quid chewing) on HNC risk, and thus, we were unable to explore this topic further in the meta-analysis.

The results from the meta-analysis showed that the inverse association between allergies and HNC was stronger among the case-control studies (meta-RR = 0.58, 95% CI: 0.44–0.78) compared to that among the cohort studies (meta-RR = 0.91, 95% CI: 0.71–1.17). All except one of the cohort studies on the association between allergies and HNC used data from medical records or clinical tests, whereas all of the case-control studies used self-reported data of allergies. Self-reported data might be more inaccurate due to recall errors associated with false memory or misinterpretation of non-allergic symptoms (*e.g.* common cold) as allergies. These recall errors were likely non-differential and would have biased the results toward the null; thus, the actual inverse association between allergies and HNC should be stronger in the absence of non-differential recall errors. Self-reported data in a case-control study can also be subject to differential recall bias. Usually, case subjects are more likely to ruminate about the possible causes of their diseases and may thus report more allergic symptoms, leading to a false positive association between allergies and HNC. However, the current meta-analysis indicated an inverse association between allergies and HNC for the case-control studies. In addition, the potential association between allergies and cancer is not well-known to the public; therefore, the inverse association between allergies and HNC among the case-control studies is unlikely due to differential recall bias. Compared to the cohort studies, the case-control studies were more likely to suffer from reverse causality, since the information on allergies was ascertained after the diagnosis of HNC and the immune function might be affected by the oncogenic process. In our original hospital-based case-control study, even when we considered allergies occurring more than 10 years before the diagnosis of cancer (or before the interview date for the controls), a strong inverse association between allergies and HNC still existed. In a hospital-based case-control by Vena et al. which considered only allergies diagnosed five years before the hospital admission, a reduced risk of HNC associated with allergies was observed among men [26]. These indicate that reverse causality might not play a major role in the inverse relationship between allergies and HNC. Although cohort studies do not suffer from recall bias or reverse causality, they are not without limitations. A major limitation of cohort studies, especially when studying a rare disease or a disease with a long latency period (*e.g.* cancer), is that there may not be enough outcomes during the follow-up period to produce a precise effect estimate [40].

An alternative method to study the association between allergies and HNC is “Mendelian Randomization”, which utilizes a functional genetic polymorphism as an instrumental variable to study the relationship between modifiable exposures and disease in observational studies [41]. Genetic polymorphisms are not subject to recall or selection biases and therefore may serve as a more objective marker. For example, interleukin-4 is a key cytokine that induces naïve T-helper cells to differentiate into T-helper 2 cells, which play a major role in the development of allergies [42]. The T allele of a promoter single nucleotide polymorphism of the interleukin-4 gene, C-589T (also known as C-590T or rs2243250), has been associated with a higher level of serum IgE and a higher risk of allergy [43]. To date, three studies have examined the relationship between rs2243250 and HNC, specifically oral cancer [44]–[46]. Two of the three studies showed an increased risk of oral cancer with either the C allele [45] or the CC genotype [46], consistent with an inverse association between allergies and HNC. However, one study reported an opposite finding with an increased risk of oral cancer associated with the TT genotype [44]. With more investigations of rs2243250 and other genetic polymorphisms associated with allergies, the true association and the underlying biological mechanism between allergies and HNC may be clarified.

The current meta-analysis suggests that the inverse association between allergies and HNC may be particularly strong for allergic rhinitis. It has been long observed that not all people with atopic tendency will develop allergic symptoms and those who develop allergies can have different symptoms with some develop asthma while others develop allergic rhinitis or different combinations of symptoms [13], [15]. Studies have detected IgE to specific allergens in the nasal washing among patients with allergic rhinitis regardless of their systemic allergy test status, suggesting the importance of local IgE production in the development of allergic rhinitis [15]. It is possible that allergic rhinitis may be an indicator for a more active local immunity in the upper aerodigestive tract against foreign substances, leading to a reduced HNC risk; however, this needs to be confirmed with further investigations.

The inverse association between allergies and HNC was present predominantly among men as indicated by the results of the current meta-analysis. While there is no obvious explanation for this observation, several HNC differences between men and women may provide clues. The incidence of HNC is higher among men than women, ranging from 1.6 times to 9.8 times higher depending on the anatomical sites and the regions of the world. Studies have shown that a higher proportion of HNC among men (74–82%) can be attributed to alcohol and cigarette compared to women (50–61%) [7], [47], [48], suggesting that HNC in women may have other causes that are less common among men. Although cigarette smoking may explain more of the HNC cases among men than among women, women appear to be more susceptible to the carcinogenic effect of cigarette. For those who smoked up to one cigarette pack per day, the HNC risk was higher in women (hazard ratio: 3.08, 95% CI: 1.77–5.36) than in men (hazard ratio: 1.26, 95% CI: 0.99–1.61) [49]. The possible differences in the causes of and the susceptibility to HNC between men and women may have contributed to their disparity in the association between allergies and HNC. More investigations on these gender differences may help establish the biological mechanism underlying the relationship between allergies and HNC.

Several limitations must be considered when interpreting the results of the current analysis. Because our case-control study is hospital-based, it is difficult to determine whether the cases and controls arise from the same source population. Our recruiting hospital is a major medical center in Southern Taiwan, and over 95% of our study subjects came from the county where the hospital is located. Even though we tried to exclude controls with diseases related to the use of alcohol, betel quid, and cigarette smoking, the possibility remains that our controls may not be representative of the background population for the cases. For the meta-analysis, studies were heterogeneous in design, patient population, disease sites, and types of allergies assessed. We explored the sources of heterogeneity by performing subgroup analyses by study design, disease sites, types of allergies, and gender. In addition, random-effect models were used to account for the heterogeneity across studies. Finally, the association between allergies and HNC was only assessed qualitatively with a binary status (yes vs. no) in the current analysis. More studies with quantitative measures of allergies (*e.g.* severity as indicated by the level of serum IgE, numbers of allergy-associated genetic variants, cytokine profiles) may provide a clearer picture to describe the relationship between allergies and HNC.

In summary, we observed an inverse association between allergies and HNC, particularly among men, in our original case-control study and meta-analysis. This suggests that immunity may play an important role to influence the risk of HNC. Future studies may investigate immune biomarkers, including cytokine profiles and genetic polymorphisms to further delineate the relationship between allergies and HNC. In addition, more studies are needed to determine whether allergies can reduce the risk HNC associated with alcohol, betel quid, or cigarette. Understanding the relationship between allergies and HNC may help devise effective strategies to reduce and treat HNC.

## Supporting Information

Table S1 **Fourteen studies (9 cohort studies and 5 case-control studies) included in the meta-analysis for the association between allergies and head and neck cancer.**
(DOC)

Checklist S1 **PRISMA Checklist.**
(DOC)
