# Supplementary material for: Allergies and Risk of Head and Neck Cancer: An Original Study plus Meta-Analysis
Source: PLoS One. 2013 Feb 1;8(2):e55138. doi: 10.1371/journal.pone.0055138 (PMC3562357; doi:10.1371/journal.pone.0055138)
Supplement: Table S1 — Fourteen studies (9 cohort studies and 5 case-control studies) included in the meta-analysis for the association between allergies and head and neck cancer. (DOC) [file pone.0055138.s001.doc]

Table S1. Fourteen studies (9 cohort studies and 5 case-control studies) included in the meta-analysis for the

association between allergies and head and neck cancer

| **9 cohort studies (Total of 727,569 subjects and 550 head and neck cancer outcomes)** | | | | | | |
| --- | --- | --- | --- | --- | --- | --- |
| **Author, year, location** | **Subject selection** | **Types of allergy assessed** | **Outcome assessment** | **Incidence ratio or standardized mortality ratio (95% confidence interval)** | **Matched or adjusted variables** | **Comments** |
| Polednak *et al.*, 1975, United States^1^ | 12,098 men born between 1850 and 1899, who were applicants for rental of a gymnasium locker at Harvard University, were recruited between 1880 and 1920. | Self-reported history of asthma:  169 men had asthma and 11,929 had no asthma | Various causes recorded on death certificates, including cancer, by June 30, 1967. One among 169 men with asthma and 47 among 11,929 men without asthma developed cancers of the buccal cavity and pharynx. | Mortality rate due to cancers of the buccal cavity and pharynx:  Asthmatics: 0.6 per 100  Non-Asthmatics: 0.4 per 100 | **Matched**  None  **Adjusted**  None | The number of subjects with asthma (n=169) was small for this cohort study and the numbers of death due to buccal and pharyngeal cancer were small (1 for asthmatics and 47 for non-asthmatics). Therefore, the result of this study could be imprecise. Cancer of the larynx was not evaluated. |
| Robinette *et al.*, 1978, United States^2^ | 9,550 asthmatic male veterans, who were alive on January 1, 1946, were identified from the medical and administrative records of the Department of Defense and the Veterans Administration.  9,550 non-asthmatic veterans from the same database matched to asthmatic veterans by age | Diagnosis of asthma on medical records | Causes of death recorded on death certificates by 1974; The numbers of death due to buccal and pharyngeal cancer : 11 for asthmatics and 10 for non-asthmatics  The number of death due to laryngeal cancer: 6 for asthmatics and 8 for non-asthmatics | Mortality rate due to cancers of the buccal cavity and pharynx:  Asthmatics: 11/9,550  Non-Asthmatics: 10/9,550  Mortality rate due to cancers of the larynx  Asthmatics: 6/9,550  Non-Asthmatics: 8/9,550 | **Matched**  Age | The numbers of death due to buccal and pharyngeal cancer (11 for asthmatics and 10 for non-asthmatics) and larynx (6 for asthmatics and 8 for non-asthmatics) were small. Therefore, the result of this study could be imprecise. |
| Källen *et al.*, 1993, Sweden^3^ | 64,346 asthmatic patients identified from the Hospital Discharge Registry of Sweden from 1969 to 1984 | Diagnosis of asthma from hospital discharge registry | Cancer of all sites recorded by the cancer registry and death registry by 1987; 84 patients developed cancer of lip-pharynx | Cancer of lip-pharynx:  0.60 (0.49-0.75) | **Adjusted:** age-and sex- standardized | Cancer of the larynx was not evaluated. |
| Vesterinen *et al.*, 1993, Finland^4^ | 77,952 asthmatic patients (35,126 men and 42,826 women) identified from the Social Insurance Institution Registers from 1970 to 1985 | Reimbursement of the costs of medication for bronchial asthma recorded on the Social Insurance Institution Registers. | Cancer of all sites recorded by the cancer registry by December 31, 1987; 20 men and 3 women developed laryngeal cancer. | Laryngeal cancer:  Men: 0.63 (0.38-0.96)  Women: 0.87 (0.18-2.54) | **Adjusted:** standardized by age, sex, period of cancer diagnosis, and follow-up period from the beginning of the entitlement to reimbursed medication | The numbers of laryngeal cancer were small (20 for men and 3 for women). Therefore, the result of this study could be imprecise. Cancers of the oral cavity and pharynx were not evaluated. |
| Eriksson *et al.*, 2005, Sweden^5^ | 13,811 (5,973 men and 7,838 women),who underwent skin prick tests for atopy from 1976 to 1999 at the Lung and Allergy Clinic of County Hospital in Halmstad, Sweden. | Atopy status (atopic, intermediate, and non-atopic) according to the skin prick tests | Diagnosis of Cancer recorded on Swedish National Cancer Registry by December 31, 1999; One patient developed pharyngeal cancer and another one developed laryngeal cancer | Pharyngeal cancer:  Intermediate atopic: 2.54 (0.06-14.1)  Atopic: 0.00 (0.00-3.63)  Laryngeal cancer:  Intermediate atopic:5.37 (0.14-29.9)  Atopic: 0.00 (0.00-16.1) |  | The numbers of laryngeal and pharyngeal cancer were too small (only 1 each) to have a sufficient statistical power for detecting a meaningful association. Cancer of the oral cavity was not evaluated. |
| Hagströmer *et al.*, 2005, Sweden^6^ | 15,666 patients with atopic dermatitis (7,635 men and 8,031 women) diagnosed between January 1, 1965 and December 31, 1999. | Atopic dermatitis recorded on the National Inpatient Register | Diagnosis of Cancer recorded on Swedish National Cancer Registry by December 31, 1999; 9 developed buccal cancer. | Buccal cancer:  1.7 (0.8-3.2) | **Adjusted:** standardized by age, sex, and calendar year | The number of buccal cancer was small (9 cases) to produce a precise estimate. Cancers of the pharynx and larynx were not evaluated. |
| Ji *et al*., 2009, Sweden^7^ | 140,425 patients hospitalized for asthma between 1965 and 2004. | Asthma diagnosis recorded in the Swedish Hospital Discharge Register | Diagnosis of cancer recorded in Swedish Cancer Registry by December 31, 2004; 115 developed upper aerodigestive tract cancer | Upper aerodigestive tract cancer: 1.14 (0.94-1.37) | **Adjusted:**  standardized by age, gender, time period, socioeconomic status, and residential area | Only hospitalized asthma patients and no outpatient asthma patients were included |
| Engkilde *et al.*, 2011, Denmark^8^ | 16,992 (6,113 men and 10,809 women), who underwent patch tests for contact allergy at the Department of Dermatology, Gentofte Hospital, Denmark between November 1984 and December 2008. 7,932 were positive for contact allergy and 8,990 were negative for contact allergy | Contact allergy as assessed by patch tests. | Diagnosis of cancer recorded by the Danish Cancer Registry; 27 of the 7,932 with contact allergy and 46 of the 8,990 without contact allergy developed cancers of the lip, oral cavity and pharynx | Cancers of the lip, oral cavity, and pharynx:  1.18 (0.73-1.92) | **Adjusted:**  sex and age | Cancer of the larynx was not evaluated. |
| Hwang *et al.*, 2011, Taiwan^9^ | 225,315 patients with allergic rhinitis (111,490 men and 113,825 women), 107,601 with asthma (54,650 men and 52,951 women), and 34,263 with atopic dermatitis (15,514 men and 18,749 women) identified from the National Health Insurance Research Database. | Diagnosis of allergic rhinitis, asthma or atopic dermatitis recorded on the National Health Insurance Claims Data. | Diagnosis of cancer recorded on the National Health Insurance Claims Data.  Number of patients developed tongue cancer:  41/225,315 with allergic rhinitis; 32/107,601 with asthma;  3/34,263 with atopic dermatitis  Number of patients developed hypopharyngeal cancer:  18/225,315 with allergic rhinitis; 19/107,601 with asthma;  3/34,263 with atopic dermatitis | Tongue Cancer:  Allergic rhinitis: 0.71 (0.51-0.97)  Asthma: 0.83 (0.57-1.17)  Atopic dermatitis: 0.53 (0.11-1.55)  Hypopharyngeal Cancer:  Allergic rhinitis: 0.62 (0.37-0.98)  Asthma: 0.83 (0.50-1.30)  Atopic dermatitis: 1.08 (0.22-3.16) | **Adjusted:**  standardized by age and sex | Cancer of larynx was not evaluated. |
| **5 case-control studies (4,017 head and neck cancer cases and 10,928 controls)** | | | | | | |
| **Author, year, location (reference)** | **Case selection** | **Control selection** | **Exposure assessment** | **Odds ratio (95% confidence interval)** | **Matched or adjusted variables** | **Comments** |
| Vena *et al.*, 1985, USA^10^ | 13,571 white cancer patients diagnosed at the Roswell Park Memorial Institute from 1957 to 1965, including 1,643 head and neck cancer cases (950 oral cancer, 325 pharyngeal cancer, and 368 laryngeal cancer). Among the 1,643 head and neck cancer cases, 1357 were men and 286 were women. | 4,039 white patients (1,562 men and 2,477 women) admitted to the same institute for non-cancer diseases, excluding those admitted for disorders of the skin and respiratory system. | Lifetime history of asthma, hay fever, hives, and eczema were ascertained by interview. | Oral cancer:  Male:  Asthma: 0.82  Hay fever: 0.49  Hives: 0.60  Other allergies: 0.53  Female  Asthma: 1.75  Hay fever: 1.72  Hives: 0.90  Other allergies: 0.85  Pharyngeal cancer:  Male:  Asthma: 0.67  Hay fever: 0.22  Hives: 0.60  Other allergies: 0.50  Female  Asthma: 1.39  Hay fever: --  Hives: 1.32  Other allergies: 0.96  Laryngeal cancer:  Male:  Asthma: 1.49  Hay fever: 0.50  Hives: 0.88  Other allergies: 0.48 | **Adjusted:**  Age and smoking | Cases and controls may not come from the same population source in this hospital-based study.  Recall-bias is a concern. |
| Petroianu *et al.*, 1995, Brazil^11^ | 400 cancer cases, including 22 cases of head and neck cancer, diagnosed at four hospitals in Belo Horizonte, Brazil. | 400 healthy controls which included students of medical school and teachers of the same institution, relatives and friends of the authors. | Self-reported history of rhinitis, skin rash and or/atopic dermatitis provoked by specific reactions to food chemicals, medications or other allergens. | % with allergies among controls: 40%  % with allergies among head and neck cancer patients: 27% | **Matched**  None  **Adjusted**  None | The number of head and neck cancer cases was very small (n=22)  Cases and controls may not come from the same population source.  Recall-bias is a concern. |
| Bosetti *et al.*, 2004, Italy^12^ | Digestive tract cancer and laryngeal cancer diagnosed in various hospitals in Italy between 1991 to 2000, including 589 cases of oral and pharyngeal cancer (512 men and 86 women) and 460 cases of laryngeal cancer (415 men and 45 women). | 4,999 patients admitted to the same hospitals as cases with acute non-neoplastic disease unrelated to known or likely risk factors for the neoplasm studied. | History of allergies was obtained by interview | Oral and pharyngeal cancer:  0.44 (0.26-0.75)  Laryngeal cancer:  0.33 (0.18-0.61) | **Adjusted:**  Age, sex, study center, years of education, body mass index, alcohol and tobacco consumption. | Recall-bias is a concern.  Cases and controls may not come from the same population source in this hospital-based study. |
| Michaud *et al.*, 2012, USA^13^ | 1,056 incident cases of head and neck cancer (765 men and 291 women) diagnosed at 9 medical facilities in the Greater Boston Area, which covers 99% of the cases in the catchment area. The recruitment had two phases: From December 1999 to December 2003 for Phase I and from October 2006 to June 2010 for Phase II. | 1,252 controls were randomly selected from the same population and frequency-matched to cases by sex, age (±3 years) and town of residence. | History of allergies was obtained by self-administered questionnaire. | Head and neck cancer:  Any allergy: 0.81 (0.67-0.98)  Asthma: 0.89 (0.66-1.22)  Oral cancer:  Any allergy: 0.98 (0.76-1.26)  Asthma: 0.90 (0.61-1.33)  Oropharyngeal cancer:  Any allergy: 0.73 (0.57-0.92)  Asthma: 0.67 (0.44-0.997)  Laryngeal cancer:  Any allergy: 0.66 (0.45-0.97)  Asthma: 0.72 (0.39-1.33) | **Matched:**  Sex, age (±3 years) and town of residence.  **Adjusted:**  Age, sex, race, smoking, alcohol consumption, education, and study phase. | Recall-bias is a concern. |
| Hsiao *et al.*, 2012, Taiwan (Current study) | 247 incident cases of head and neck cancer (165 oral cancer, 57 oropharyngeal cancer, and 25 laryngeal cancer) diagnosed in the departments of otolaryngology and stomatology at a medical center in Tainan City, Taiwan between September 1, 2010 and June 30, 2012 | 238 controls who underwent surgery for non-cancerous conditions in the department of otolaryngology in the same hospital. | History of allergies was obtained by in-person interview. | Head and neck cancer:  Any allergy: 0.41 (0.27-0.62)  Allergic rhinitis: 0.32 (0.20-0.52)  Skin allergy: 0.67 (0.38-1.17)  Food allergy: 0.63 (0.30-1.31)  Drug allergy: 0.44 (0.21-0.92)  Asthma: 0.26 (0.09-0.73)  Oral cancer:  Any allergy: 0.36 (0.22-0.57)  oropharyngeal cancer:  Any allergy: 0.49 (0.25-0.96)  Laryngeal cancer:  Any allergy: 0.48 (0.19-1.18) | **Matched:**  Age and sex (±5 years)  **Adjusted:**  Age, sex, education, consumption of alcohol, betel quid and cigarette. | Recall-bias is a concern.  Cases and controls may not come from the same population source in this hospital-based study. |

**References**

1. Polednak AP. Letter: Asthma and cancer mortality. Lancet 1975;2:1147-8.

2. Robinette CD, Fraumeni JF, Jr. Asthma and subsequent mortality in World War II veterans. Journal of chronic diseases 1978;31:619-24.

3. Kallen B, Gunnarskog J, Conradson TB. Cancer risk in asthmatic subjects selected from hospital discharge registry. Eur Respir J 1993;6:694-7.

4. Vesterinen E, Pukkala E, Timonen T, Aromaa A. Cancer incidence among 78,000 asthmatic patients. Int J Epidemiol 1993;22:976-82.

5. Eriksson NE, Mikoczy Z, Hagmar L. Cancer incidence in 13811 patients skin tested for allergy. J Investig Allergol Clin Immunol 2005;15:161-6.

6. Hagstromer L, Ye W, Nyren O, Emtestam L. Incidence of cancer among patients with atopic dermatitis. Arch Dermatol 2005;141:1123-7.

7. Ji J, Shu X, Li X, Sundquist K, Sundquist J, Hemminki K. Cancer risk in hospitalised asthma patients. Br J Cancer 2009;100:829-33.

8. Engkilde K, Thyssen JP, Menne T, Johansen JD. Association between cancer and contact allergy: a linkage study. BMJ Open 2011;1:e000084.

9. Hwang CY, Chen YJ, Lin MW, Chen TJ, Chu SY, Chen CC, Lee DD, Chang YT, Wang WJ, Liu HN. Cancer risk in patients with allergic rhinitis, asthma and atopic dermatitis: a nationwide cohort study in Taiwan. International journal of cancer 2012;130:1160-7.

10. Vena JE, Bona JR, Byers TE, Middleton E, Jr., Swanson MK, Graham S. Allergy-related diseases and cancer: an inverse association. American journal of epidemiology 1985;122:66-74.

11. Petroianu A, Chaves DN, De Oliveira O, Jr. Comparative incidence of allergy in the presence or absence of cancer. The Journal of international medical research 1995;23:358-63.

12. Bosetti C, Talamini R, Franceschi S, Negri E, Giacosa A, La Vecchia C. Allergy and the risk of selected digestive and laryngeal neoplasms. Eur J Cancer Prev 2004;13:173-6.

13. Michaud DS, Langevin SM, Eliot M, Nelson HH, McClean MD, Christensen BC, Marsit CJ, Kelsey KT. Allergies and risk of head and neck cancer. Cancer Causes Control 2012;23:1317-22.
